# Anti-Hypertensive Medication Use, Soluble Receptor for Glycation End Products and Risk of Pancreatic Cancer in the Women’s Health Initiative Study

**DOI:** 10.3390/jcm7080197

**Published:** 2018-08-02

**Authors:** Zhensheng Wang, Donna L. White, Ron Hoogeveen, Liang Chen, Eric A. Whitsel, Peter A. Richardson, Salim S. Virani, Jose M. Garcia, Hashem B. El-Serag, Li Jiao

**Affiliations:** 1 Section of Epidemiology and Population Sciences, Department of Medicine, Baylor College of Medicine, Houston, TX 77030, USA; zhenshew@bcm.edu; 2 Section of Gastroenterology and Hepatology, Department of Medicine, Baylor College of Medicine, Houston, TX 77030, USA; dwhite1@bcm.edu (D.L.W.); liangc@bcm.edu (L.C.); hasheme@bcm.edu (H.B.E.-S.); 3 Center for Innovations in Quality, Effectiveness and Safety (IQuESt), Michael E. DeBakey VA Medical Center, Houston, TX 77030, USA; peter.richardson2@va.gov (P.A.R.); virani@bcm.edu (S.S.V.); 4 Section of Health Services Research, Department of Medicine, Baylor College of Medicine, Houston, TX 77030, USA; 5 Section of Atherosclerosis and Vascular Medicine, Department of Medicine, Baylor College of Medicine, Houston, TX 77030, USA; ronh@bcm.edu; 6 Texas Medical Center Digestive Disease Center, Houston, TX 77030, USA; 7 Dan L. Duncan Cancer Center at Baylor College of Medicine, Houston, TX 77030, USA; 8 Center for Translational Research on Inflammatory Diseases (CTRID), Michael E. DeBakey VA Medical Center, Houston, TX 77030, USA; 9 Departments of Epidemiology and Medicine, Gillings School of Global Public Health and School of Medicine, University of North Carolina at Chapel Hill, Chapel Hill, NC 27599, USA; eric_whitsel@med.unc.edu; 10 Geriatric Research Education and Clinical Center (GRECC), Puget Sound Department of Veterans Affairs Medical Center, Seattle, WA 98108, USA; Jg77@uw.edu; 11 Section of Endocrinology, Department of Medicine, University of Washington, Seattle, WA 98195, USA

**Keywords:** pancreatic neoplasm, pharmacoepidemiology, hypertension, calcium channel blocker, inflammation, risk factor, sRAGE

## Abstract

Pancreatic cancer is the fourth leading cause of cancer death. Soluble receptor for glycation end products (sRAGE), which is modulated by anti-hypertensive (HT) medications, has been inversely associated with pancreatic cancer. However, the association between commonly used anti-HT medications and risk of pancreatic cancer is unknown. A total of 145,551 postmenopausal women from the Women Health Initiative (WHI) Study were included in analysis. Use of angiotensin converting enzyme inhibitors (ACEi), β-blockers, calcium channel blockers (CCBs) and diuretics was ascertained at baseline (1993–1998). Baseline sRAGE levels were measured among a subset of 2104 participants using an immunoassay. Multivariable Cox proportional hazard regression model was performed to estimate hazard ratios (HRs) and its 95% confidence intervals (CIs) for pancreatic cancer in association with anti-HT medications. Increased risk of pancreatic cancer was found among users of short-acting CCB (HR = 1.66, 95% CI: 1.20–2.28) and long-term (≥3 years) users of short-acting CCB (HR = 2.07, 95% CI: 1.42–3.02) compared to users of other anti-HT medications. Average sRAGE levels were lower in short-acting CCB users than users of other anti-HT medications (1173 versus 1454 pg/mL, *p* = 0.038). Non-statistically significant reduced risk of pancreatic cancer was found among users of β-blockers (HR = 0.80, 95% CI: 0.60–1.07). Average sRAGE levels were higher in β-blockers users than users of other anti-HT medications (1692 versus 1454 pg/mL, *p* > 0.05). Future studies are warranted to confirm these findings and elucidate potential mechanisms by which anti-HT medications influence development of pancreatic cancer.

## 1. Introduction

Pancreatic cancer is the fourth leading cause of cancer-related death in the United States [1]. Established modifiable risk factors for pancreatic cancer include cigarette smoking, heavy alcohol consumption, obesity and long-standing type 2 diabetes [2]. The incidence of pancreatic cancer is expected to rise in the aging population where hypertension is one of the most common comorbidities with an estimated prevalence of 65% among population aged over 60 in the U.S. [3]. It will be of potential public health significance to establish an association between commonly used medications and pancreatic cancer development [4]. 

Multiple anti-HT medications have been extensively studied in relation to risk of developing cancer. A recent meta-analysis of 70 clinical trials found no significant associations between angiotensin II receptor blockers (ARBs), angiotensin-converting enzyme inhibitor (ACEi), β-blockers, calcium-channel blockers (CCBs), or diuretics and overall risk of developing cancer [5]. However, the association between anti-HT medications use and risk of developing pancreatic cancer has not been examined in large-scale population-based studies. 

We previously showed a significant inverse association between soluble receptor for advanced glycation end products (sRAGE) and risk of incident pancreatic cancer [6,7]. The anti-inflammatory sRAGE is thought to mitigate the inflammatory response induced by ligation of receptor for AGE (RAGE) and its ligands [8]. Anti-HT medications, including ACEi [9,10], β-blocker [11] and CCBs [10,12], have been shown to increase sRAGE concentrations, decrease formation of advanced glycation end-products (AGEs), or dampen pro-inflammatory RAGE signaling pathway. It is plausible that these anti-HT medications can potentially lower risk of pancreatic cancer by modulating RAGE signaling. 

Therefore, we examined the associations between the use of ACEi, β-blockers, CCBs and diuretics and risk of developing incident pancreatic cancer in a large prospective cohort. We hypothesized that use and long-term use of ACEi, β-blockers, CCBs and diuretics are associated with a reduced risk of pancreatic cancer. Additionally, we explored the association between sRAGE levels and the anti-HT medication use in a subset of study participants for a mechanistic explanation. 

## 2. Methods

### 2.1. Study Population

The Women’s Health Initiative (WHI) Study is a long-term prospective cohort study in the U.S. aiming to investigate preventative strategies against heart diseases, breast and colorectal cancers and osteoporotic fractures. Study participants were either assigned into the randomized clinical trial (CT) or observational study (OS) [13]. The main exclusion criteria for OS and CT study are: (1) having conditions predicative of survival time <3 years; (2) having conditions (such as alcoholism, drug dependency, mental illness and dementia influencing study participation and adherence; (3) participation in other clinical trials [14]. A total of 161,808 postmenopausal women (aged 50 to 79 years) were recruited from 40 clinical centers nationwide during 1993–1998. Participants with prevalent cancer other than non-melanoma skin cancer at baseline (*n* = 16,255) were excluded from the current analysis. We included a total of 145,551 postmenopausal women from the WHI-OS and CT for the present analysis. To study the long-term effect of interventions on health outcomes, two extension studies were initiated in 2005 (extension study I) and 2010 (extension study II) with a re-consent rate of 72% and 59%, respectively. The study protocol was approved by the Institutional Review Board of both Baylor College of Medicine (BCM) and the Michael E. DeBakey VA Medical Center in Houston, TX, USA.

### 2.2. Ascertainment of Medication Use

At baseline interview, all participants were asked to bring currently used medications and supplement bottles used for at least two weeks. The interviewers entered product and generic name, dosage form, medic strength, therapeutic class and ingredients directly from the containers into a database that assigned drug codes using Medi-Span software (First DataBank, Inc., San Bruno, CA, USA), a pharmaceutical reference database. The study participants also reported the duration of medication use.

Types of medication were determined based on the 6-digit medication therapeutic class code (TCC) as following: ACEi (361000, 369915, 369918, 369985, 964642, 965068, or 966458), β-blockers (330000, 331000, 332000, 369920, 369925, 369988 or 966442, CCBs (340000, 369915, 369925, 369930, 409925 or 964858) and diuretics (370000, 373000, 376000 or 379900) [15]. These codes included the combined use of anti-HT medications as well as monotherapy. Short-acting CCBs were defined as (1) any non-continuous release (CR)/sustained release (SR)/extended release (ER/XR/XT) formulation, or (2) Nisoldipine. Long-acting CCB was defined as (1) any CR, SR, ER, XR or XT formulations, or (2) Amlodipine besylate or Felodipine. Dihydropyridine (DHP) CCBs included Amlodipine, Felodipine, Isradipine, Nicardipine, Nifedipine and Nisoldipine. Non-dihydropyridine (NDHP) CCBs included Verapamil, Diltiazem, Mibefradil and Bepridil. 

### 2.3. Ascertainment of Pancreatic Cancer

Pancreatic cancer cases were ascertained through self-administered questionnaires semi-annually for CT participants and annually for OS participants. Pancreatic cancer was defined using the ICD-O-2 code of C25.0–C25.4, C25.7–C25.9. All the cases were locally and centrally adjudicated by trained physicians using pathology/cytology report, operative report, hospital discharge summary, outpatient, day surgery or short stay record [16]. We ascertained pancreatic cancer cases through 29 August 2014 for this analysis. 

### 2.4. Data Collection

Information on age, race/ethnicity, education, income, marital status, smoking habit, alcohol consumption, medical history and recreational physical activity at baseline were obtained by recruitment survey. Height, weight, hip and waist circumference were measured by trained clinic staff at the first clinic visit [17]. History of hypertension and type 2 diabetes were self-reported regardless of oral medication treatment [17]. Food consumption in the past three months was assessed using a self-administered food frequency questionnaire (FFQ) [17].

### 2.5. Measurement of sRAGE

In two previous nested case-control studies within the WHI, we measured baseline serum levels of sRAGE [7,18] using human sRAGE Quantikine ELISA kit (R&D System Inc., Minneapolis, MN, USA) at BCM. Our study included 489 pancreatic cancer cases that were ascertained until August 2014 and 977 controls that were individually matched to each case according to age at baseline (±3 years), ethnicity, baseline blood draw date (±6 months), OS enrollment (yes/no), HT trial arm (yes/no), calcium and vitamin D trial arm (yes/no), hysterectomy at baseline (yes/no) and study center [18]. In the other study on colorectal cancer, we measured sRAGE levels of among 638 cancer-free controls [7]. Because the ELISA for sRAGE is a highly reproducible assay, we did not find the sRAGE measured in two studies to be systematically different. In addition, we observed the same association between sRAGE and anti-HT medication in 977 and 638 controls respectively, we presented the association by merging the two analyses together. 

### 2.6. Statistical Analysis

Because we observed that the CCBs use to be associated with increased risk of pancreatic cancer, baseline demographic characteristics, lifestyle factors, dietary factors and selected medical history were compared according to CCB use, use of non-CCBs medications (ACEi, diuretics or β-blockers) and non-use of any anti-HT medications at baseline. 

Cox proportional hazard regression model was performed to assess the association between hypertension and anti-HT medication use and risk of developing incident pancreatic cancer. Due to low rate of monotherapy in study participants, we included both monotherapy and polytherapy in defining medication use. We used two reference groups in data analysis: (1) non-users of any anti-HT medications; (2) users of anti-HT medications other than the one under study. The number of years of follow-up was calculated from the baseline recruitment until pancreatic cancer diagnosis, death, loss to or end of follow-up (29 August 2014), whichever occurred first. Test for proportional hazard assumption was conducted based on the statistical significance of correlation between Schoenfeld residuals and ranked survival time. We found no violation of the proportional hazard assumptions. Adjusted variables were selected if they were: (1) established risk factor for pancreatic cancer based on literature review; or (2) associated with risk of pancreatic cancer in the univariable analysis (*p* < 0.25) and with the use of medications (*p* < 0.25). Final model included age, race/ethnicity (White, Black, American Indian or Alaskan Native, Asian or Pacific Islander, Hispanics), BMI (<25, 25–<30, ≥30 kg/m^2^), smoking status (never, past or current smokers), alcohol consumption more than 3 drink/day (yes vs. no), self-reported type 2 diabetes (yes vs. no) and hypertension (yes vs. no). Further adjustment of other variables such as OS/CT assignment, family history of cancer, physical activity, or saturated fat intake did not meaningfully alter the association. The same set of covariates (except for hypertension) was adjusted in the multivariable model in evaluating the association between hypertension and risk of pancreatic cancer. Competing risk of non-pancreatic cancer deaths were further accounted for by using the Fine and Gray model [19]. Restricted analysis was also performed among subjects with self-reported hypertension at baseline.

Because we observed the association between CCB use and risk of pancreatic cancer, we further examined whether the duration and type of CCB use (short- and long-acting CCBs, DHP- and non-DHP CCB) were associated with risk of pancreatic cancer. Among users of CCBs, duration of use was dichotomized as <3 years (median cutoff for users) versus ≥3 years. Duration of use was also included in the model as a continuous variable to test for the linear trend. Additional stratified analyses were performed according to WHR (< or ≥0.80), BMI (<25, 25–<30, or ≥30 kg/m^2^), cardiovascular diseases, type 2 diabetes and sRAGE levels (using median among controls as the cutoff). Time-varying Cox regression by using years 3 (OS and CT) and 6 (CT only) data on anti-HT medication use status were performed to further assess the association between over-time use and risk of pancreatic cancer [20].

Among 1615 controls who had sRAGE levels measured, anti-HT medication use information was documented for 1522 participants. Furthermore, a total of 1388 participants reported non-use or use of only one anti-HT medications. Least square means of sRAGE concentration were compared across non-users of any anti-HT medications and users of single anti-HT medication by adjusting for age, BMI, smoking status, alcohol consumption ≥3 drinks/day and self-reported hypertension. We reported sRAGE levels by both the monotherapy (*n* = 1388) and monotherapy plus polytherapy (*n* = 1522) among controls. Sidak multiple comparison adjustment was applied [21]. In 489 cases and 1522 controls, we separately evaluated the association between anti-HT medication use and risk of pancreatic cancer using Cox regression model with sRAGE as an additional covariate in its continuous form. 

To address the concern over potential reverse causality the observations between anti-HT medication use and pancreatic cancer risk, we conducted sensitivity analysis by excluding all participants with less than two years of follow-up. We also performed all the analysis among the participants who were diagnosed with hypertension at baseline. To control for residual confounding, we alternatively conducted propensity score matching on CCB use and other anti-HT medications use by performing logistic regression. For each CCB user, we matched with 5 non-CCB anti-HT medication users if absolute difference of their propensity score was ≤0.01 [22]. 

All analyses were conducted using SAS 9.4 (SAS Inc., Cary, CA, USA) software. Two-sided *p* value less than 0.05 was considered statistically significant.

## 3. Results

### 3.1. Baseline Characteristics of Participants

With an average of 13.8 (standard deviation = 4.8) years of follow-up, a total of 841 incident pancreatic cancer cases were ascertained. Compared with non-users of any anti-HT medications, users of CCBs were more likely to be older, in a minor racial/ethnic category, less educated, current smokers, obese, inactive and to have hypertension, pancreatitis and type 2 diabetes (Table 1). 

### 3.2. Anti-HT Medications and Risk of Pancreatic Cancer

Overall, hypertension at baseline was associated with moderately increased risk of pancreatic cancer (multivariable HR = 1.18, 95% CI: 1.02–1.36). A 40% increased risk of pancreatic cancer was found among users of CCBs compared with users of any other anti-HT medications. The association became slightly attenuated when compared with non-users of any anti-HT medications. Non-statistically significant inverse associations were found for the use of ACEi, β-blocker, or diuretics with risk of pancreatic cancer (Table 2). The associations did not alter significantly after restricting to participants with diagnosed hypertension at baseline (Supplementary Table S1). After propensity score matching was performed, 20.8% of CCB users were matched with 40.0% of other anti-HT medication users. CCB use was still associated with increased risk of pancreatic cancer compared with users of other anti-HT medications (HR = 1.52, 95% CI: 0.93–2.49) although not statistically significant. No association was found between over-time CCB use (baseline plus year 3 and 6 follow-up) and risk of pancreatic cancer (HR = 1.30, 95% CI: 0.90–1.86) compared with users of any other anti-HT medications in the time-varying analysis.

### 3.3. Duration of CCB Use and Risk of Pancreatic Cancer

Furthermore, we found only the use of short-acting CCBs was associated with an increased risk of pancreatic cancer compared with the use of any other anti-HT medications. In addition, long-term use of short-acting CCBs (≥3 years) had a 107% (HR = 2.07, 95% CI: 1.42–3.02) higher risk of pancreatic cancer (Table 3). Further analysis showed that the use of NDHP but not DHP, was associated with risk of pancreatic cancer (Supplementary Table S2).

### 3.4. sRAGE, Anti-HT Medications and Risk of Pancreatic Cancer

**Figure 1:**
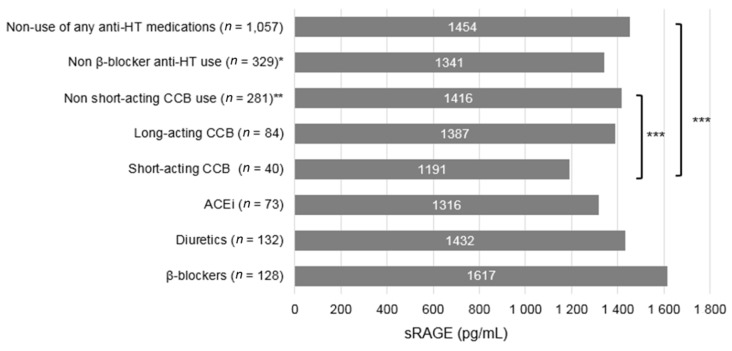
Average serum sRAGE level by anti-HT medication use among controls in the WHI Study (*n* = 1388). ACEi = Angiotensin converting enzyme inhibitor; CCB = Calcium channel blocker; DHP = Dihydropyridine; NDHP = Non-dihydropyridine; sRAGE = soluble receptor for advanced glycation end products. * Including ever use of ACEi, diuretics, or CCB. ** Including ever use of ACEi, diuretics, β-blockers, or long-acting CCB. *** *p* < 0.05, Sidak multiple comparison adjustment applied to compute *p*-value based on model adjusted for age at baseline, BMI (<25, 25–< 30 and ≥30 kg/m^2^), smoking status (never, former and current), alcohol consumption (3 drinks/day and ≥3 drinks/day), self-reported type 2 diabetes at baseline (yes or no) and self-reported hypertension at baseline (yes or no).

The average sRAGE levels were significantly lower in short-acting CCB users than non-users of any anti-HT medications (*p* = 0.021) and users of other anti-HT medications (*p* = 0.038) (Figure 1). The sRAGE level for the monotherapy of each anti-HT medications did not differed significantly (Supplementary Figure S1). In the stratified analysis by sRAGE levels, a stronger association between the CCB use and pancreatic cancer risk was observed only among women with below median sRAGE level when compared with both users of other anti-HT medications (*p* for interaction = 0.26) and non-users of any anti-HT medications (*p* for interaction = 0.12) (Table 4). We observed the same interaction effect between sRAGE and short acting CCB although the analysis was underpowered. Nevertheless, adjustment of sRAGE did not significantly change the association between short acting CCBs use and risk of pancreatic cancer.

No statistically significant interaction was found for CCB use with BMI, WHR, cardiovascular disease or diabetes (Supplementary Table S3). In the sensitivity analysis, the associations for each of anti-HT medications remained after excluding pancreatic cancers identified in the initial two years of follow-up (Supplementary Table S4). The association between other anti-HT medication use and risk of pancreatic cancer also remained the same. 

## 4. Discussion

In this large prospective cohort of postmenopausal women in the U.S., we hypothesized that the anti-HT medications that hamper RAGE inflammatory signaling would be associated with reduced risk of pancreatic cancer. Consistent with our hypothesis, we did observe a statistically non-significant inverse association between β-blockers use and risk of pancreatic cancer. However, contrary to our hypothesis, we found that short-acting CCBs use was associated with an increased risk of pancreatic cancer, especially among long-term users. Furthermore, we found that β-blockers use was associated with non-statistically significant higher levels of sRAGE and short-acting CCBs use was associated with statistically significant lower levels of sRAGE compared with the use of other anti-HT medications. The association between CCBs use and short-acting CCBs use and pancreatic cancer also differed by sRAGE levels. 

CCBs, also known as calcium antagonists, result in lower blood pressure by preventing calcium from entering vascular smooth muscle and thus relax and dilate blood vessels [23]. Previous cell and animal studies found that CCBs can prevent pancreatic cancer development [24] and retard pancreatic cancer progression [25,26]. An in vitro study also found that CCBs can lower the mRNA expression levels of RAGE and block AGE-induced RAGE gene induction [12]. Therefore, we would expect an inverse association between CCBs use and risk of pancreatic cancer.

However, we observed a positive association between short-acting and NDHP CCB use and risk of pancreatic cancer. A significantly lower sRAGE level was also found among short-acting CCB users, compared with women who used other anti-HT medications. sRAGE could mitigate the inflammatory response by blocking RAGE-ligand binding [8]. Our previous study showed the significant inverse association between sRAGE and risk of pancreatic cancer [7]. It is unknown whether the antagonism of the calcium channel reduced sRAGE release to circulation. Furthermore, the positive association between short-acting CCB use and risk of pancreatic cancer was not seen among those with higher levels of sRAGE. It is possible that the anti-inflammatory effect of sRAGE may counteract the detrimental effect of CCBs when circulating sRAGE levels were high. In our study population, the increased risk among NDHP CCB users could be mainly due to the use of short-acting CCBs because approximately 80% of NDHP CCBs were categorized as short-acting CCBs. 

Although β-blocker has been shown to protect against pancreatic cancer development by arresting pancreatic cancer cell growth and inducing apoptosis as well as been shown to increase sRAGE levels [27], we did not observe a significant inverse association between use of β-blocker and risk of pancreatic cancer. Additional studies are needed to confirm the observed non-significant inverse association between β-blocker and risk of incident pancreatic cancer. Unlike a previous study conducted in diabetic mice [28], we did not observe increased sRAGE levels among ACEi users. In our study, ACEi and diuretics were not associated with risk of developing pancreatic cancer. Likewise, a previous nested case-control study in UK did not find the association between status and the duration of ACEi use and risk of pancreatic cancer [29]. 

CCBs was approved as a first line treatment medication for hypertension by U. S. Food and Drug Administration (FDA) in 1989 [30,31]. At the baseline of the WHI study 1993–1998, CCBs were the most commonly used monotherapy for hypertension management [32]. Our study found potential long-term detrimental effect of short-acting CCB use as a potential novel risk factor for pancreatic cancer in postmenopausal women. Although the use of short-acting CCBs declines through the years since their multiple side effects reported in late 1990s [33], its use is still lingering in clinical practice [34]. Given many anti-HT medications are currently available, our study suggested short-acting CCBs should not be used for routine care for patients with hypertension. 

The strengths of this study included the long follow-up time among a large cohort of postmenopausal women, complete ascertainment of cases, comprehensive evaluation of confounding factors, detailed record of medication use before baseline, a robust research approach including the examination of CCBs by formulations and potential mechanistic insight into our observations. Nevertheless, our study had several limitations. The findings may not be generalizable to men, to pre-menopausal women, or to post-menopausal women that substantially differ from the U.S. in prevalence of underlying risk factors for pancreatic cancer like diabetes and obesity and with different rates of hypertension and use of CCBs. Also, the data on sRAGE were available only for a subset of participants, making this mechanistic insight novel but exploratory. The finding of the excess risk particularly in the subgroup of women using short-acting CCBs was based on a smaller sample size requiring qualified interpretation and need for replication in other larger studies. The time-varying analysis and propensity score analyses in general supported the positive association between CCB use and risk of pancreatic cancer. However, due to reduced use of CCBs in the study population (33% at baseline, 22% three years after baseline in CT and OS and 20% six years after baseline in CT), the HR generated from time varying analysis was not statistically significant. Because the monotherapy was uncommon, we were not able to examine the association by monotherapy due to smaller sample size. Likewise, the ARB use was uncommon in our study population (3.7%) and its association with pancreatic cancer was thus not evaluated. Lastly, the possibility of confounding effects related to prevalent use of CCBs, indication/contraindication to use of short-acting CCBs, and/or other unrecognized risk factors cannot be excluded [35]. 

## 5. Conclusions

It is important to manage hypertension to prevent major adverse health consequences. We found that the use of commonly prescribed anti-HT medications was not significantly associated with risk of pancreatic cancer. Short-acting CCBs may potentially be a risk factor for pancreatic cancer in post-menopausal women. Further studies are needed to understand the common pathogenic mechanism shared by hypertension and pancreatic cancer such as RAGE signaling.

## Figures and Tables

**Table 1:** Selected baseline characteristics by anti-hypertensive (HT) medication use status among postmenopausal women in the Women Health Initiative Study (*n* = 145,551).

Characteristics Mean (SD) or %	CCB Use	Use of Non-CCB Anti-HT Drugs	Non-Use of Any Anti-HT Drugs
	(*n* = 14,117)	(*n* = 27,991)	(*n* = 103,443)
Age, years	65.4 (7.0)	64.6 (7.0)	62.3 (7.1)
Non-Hispanic white, %	72.2	81.5	84.1
Education status, %			
<high school	2.5	1.7	1.5
High school but no college	38.6	35.8	28.9
College or above	58.9	62.5	69.6
Smoking status, %			
Never	49.7	51.4	50.7
Former	42.4	41.7	40.9
Current	6.4	5.6	7.3
Missing	1.5	1.3	1.1
Alcohol, ≥3 drinks/day, %	9.6	10.2	12.3
BMI, kg/m^2^	30.1 (6.4)	29.8 (6.4)	27.2 (5.5)
BMI in kg/m^2^, %			
<25	22.8	24.2	40.3
25–<30	33.2	33.8	34.9
≥30	44.0	42.0	24.8
Waist to hip ratio	0.84 (0.08)	0.83 (0.08)	0.80 (0.08)
Recreational physical activity, MET-h	10.1 (12.1)	10.7 (12.4)	13.1 (14.2)
Diagnosed Hypertension, %	89.6	82.0	12.9
Pancreatitis, %	1.1	0.9	0.6
Self-report type 2 diabetes, %	13.5	10.3	3.5
Family history of cancer, %	61.1	62.3	63.6
Total fat, g/1000 cal	36.6 (9.4)	37.0 (9.2)	36.1 (9.3)
Saturated fat, g/1000 cal	12.0 (3.6)	12.2 (3.6)	12.1 (3.7)
Red meat, servings/day	0.73 (0.66)	0.74 (0.62)	0.68 (0.58)
Clinical trial assignment, %	44.4	44.8	44.4
Anti-HT medication type, %			
Short-acting CCBs	30.8	-	-
Long-acting CCBs	69.8	-	-
Dihydropyridine CCBs	74.9	-	-
Non-dihydropyridine CCBs	25.4	-	-
ACEi	13.4	33.8	-
β-blockers	11.6	34.4	-
Diuretics	30.6	54.3	-

ACEi = Angiotensin converting enzyme inhibitor; BMI = Body mass index; CCB = Calcium channel blocker; MET = Metabolic equivalent.

**Table 2:** Baseline antihypertensive medication use status in association with risk of incident pancreatic cancer.

Medication Ever Use (Cases/P-yrs)	Reference Groups (Cases/P-yrs)	HR (95% CI) ^1^	HR (95% CI) ^2^	HR (95% CI) ^3^
ACEi (66/143,598)	Use of other anti-HT meds ^4^ (205/399,060)	0.92 (0.70–1.21)	0.88 (0.66–1.16)	0.86 (0.66–1.14)
	Non-use of any anti-HT meds (570/1,454,994)	1.06 (0.83–1.38)	0.91 (0.67–1.23)	0.86 (0.64–1.16)
β-blockers (61/147,061)	Use of other anti-HT meds ^4^ (210/395,597)	0.77 (0.58–1.03)	0.80 (0.60–1.06)	0.80 (0.60–1.07)
	Non-use of any anti-HT meds (570/1,454,994)	0.94 (0.72–1.22)	0.85 (0.64–1.14)	0.82 (0.61–1.11)
CCBs (114/175,149)	Use of other anti-HT meds ^4^ (157/367,343)	1.48 (1.16–1.88)	1.44 (1.13–1.84)	1.40 (1.10–1.78)
	Non-use of any anti-HT meds (570/1,454,994)	1.45 (1.19–1.78)	1.28 (1.00–1.64)	1.20 (0.94–1.56)
Diuretics (124/248,454)	Use of other anti-HT meds ^4^ (147/294,204)	0.99 (0.78–1.26)	0.98 (0.77–1.24)	0.94 (0.74–1.20)
	Non-use of any anti-HT meds (570/1,454,994)	1.13 (0.93–1.38)	0.99 (0.78–1.26)	0.94 (0.73–1.20)

ACEi = Angiotensin converting enzyme inhibitor; CCB = Calcium channel blocker; P-yrs = Person-years. ^1^ Adjusted for age. ^2^ Adjusted for age at baseline, race/ethnicity (non-Hispanic White, Black, Hispanic, Asian or Pacific Islander and American Indian/Alaska Native), BMI (<25, 25–<30, ≥30 kg/m^2^), smoking status (never, former, current), alcohol consumption (<3 drinks/day and ≥3 drinks/day), self-reported type 2 diabetes at baseline (yes or no) and self-reported hypertension at baseline (yes or no). ^3^ Competing risks model with same covariates in ^2^. ^4^ Use of other anti-HT medications (not including the one under study).

**Table 3:** Use and duration of use of long-acting and short-acting CCBs in association with risk of incident pancreatic cancer.

Medication Use	Cases/P-yrs	HR (95% CI) ^1^	HR (95% CI) ^2^
Use of other anti-HT drugs ^3^	201/419,944	1.00 (ref.)	1.00 (ref.)
Long-acting CCBs ever use	70/122,713	1.14 (0.87–1.51)	1.12 (0.85–1.47)
Long-acting CCBs use <3 years	35/60,987	1.16 (0.81–1.67)	1.14 (0.79–1.63)
Long-acting CCBs use ≥3 years	35/61,726	1.12 (0.78–1.61)	1.10 (0.77–1.58)
*p* trend		0.810	0.652
Use of other anti-HT drugs ^3^	226/488,958	1.00 (ref.)	1.00 (ref.)
Short-acting CCBs ever use	45/53,700	1.73 (1.25–2.38)	1.66 (1.20–2.28)
Short-acting CCBs use <3 years	14/24,916	1.20 (0.70–2.06)	1.15 (0.67–1.97)
Short-acting CCBs use ≥3 years	31/28,784	2.16 (1.48–3.15)	2.07 (1.42–3.02)
*p* trend		0.004	<0.001

CCB = Calcium channel blocker; P-yrs = Person-years; sRAGE = soluble receptor for advance glycation end products. ^1^ Adjusted for age at baseline, race/ethnicity (White (non-Hispanic), Black, Hispanic, Asian or Pacific Islander and American Indian/Alaska Native), BMI (<25, 25­–<30 and ≥30 kg/m^2^), smoking status (never, former and current), alcohol consumption (<3 drinks/day and ≥3 drinks/day), self-reported type 2 diabetes at baseline (yes or no) and self-reported hypertension at baseline (yes or no). ^2^ Competing risks model with same covariates in ^1^. ^3^ Use of other anti-HT drugs include all the rest anti-HT medications studied other than the one of interest.

**Table 4:** Baseline CCB use status and risk of incident pancreatic cancer among participants with sRAGE measurements (*n* = 1522).

CCB Ever Use (Cases/P-yrs)	Reference Groups (Cases/P-yrs)	HR (95% CI) ^1^	HR (95% CI) ^2^	*p*-Interaction
All (61/2284)	Use of other anti-HT drugs ^3^ (90/5260)	1.41 (1.00–1.97)	1.38 (0.98–1.94)	0.26
	Non-use of any anti-HT drugs (338/19,109)	1.12 (0.79–1.59)	1.11 (0.78–1.58)	0.12
sRAGE < 1346 pg/mL (45/1298)	Use of other anti-HT drugs ^3^ (57/2783)	1.63 (1.08–2.46)	1.64 (1.08–2.48)	
	Non-use of any anti-HT drugs (184/8939)	1.58 (1.01–2.46)	1.59 (1.02–2.48)	
sRAGE ≥ 1346 pg/mL (16/986)	Use of other anti-HT drugs ^3^ (33/2476)	1.02 (0.54–1.92)	1.01 (0.54–1.91)	
	Non-use of any anti-HT drugs (154/10,169)	0.63 (0.34–1.18)	0.63 (0.34–1.18)	

P-yrs = Person-years; sRAGE = soluble receptor for advance glycation end products. ^1^ Competing risk model adjusted for age at baseline, race/ethnicity (non-Hispanic white, Black, Hispanic, Asian or Pacific Islander and American Indian/Alaska Native), BMI (<25, 25–< 30 and ≥30 kg/m^2^), smoking status (never, former and current), alcohol consumption (<3 drinks/day and ≥3 drinks/day), self-reported type 2 diabetes at baseline (yes or no) and self-reported hypertension at baseline (yes or no). ^2^ Adjusted for sRAGE (pg/mL) in addition to covariates in ^1^. ^3^ Use of other anti-HT drugs (not including the one under study).

## Supplementary Materials

The following are available online at http://www.mdpi.com/2077-0383/7/8/197/s1, Table S1: Baseline antihypertensive medication use status in association with risk of incident pancreatic cancer among subjects with self-report hypertension, Table S2: The duration of use of DHP- and non-DHP CCBs in association with risk of incident pancreatic cancer, Table S3: Baseline CCB use and risk of incident pancreatic cancer by potential effect modifiers in the WHI Study, Table S4: Duration of CCB use at baseline and risk of incident pancreatic cancer after excluding first 2 years of follow-up, Figure S1: Average serum sRAGE level by single anti-HT medication use among controls in the WHI Study (*n* = 1346).
