# Supplementary material for: Anti-Hypertensive Medication Use, Soluble Receptor for Glycation End Products and Risk of Pancreatic Cancer in the Women’s Health Initiative Study
Source: J Clin Med. 2018 Aug 2;7(8):197. doi: 10.3390/jcm7080197 (PMC6111748; doi:10.3390/jcm7080197)
Supplement: Supplementary file 1 [file jcm-07-00197-s001.pdf]

Supplementary Table S1. Baseline antihypertensive medication use status in association with risk of incident pancreatic cancer among subjects with self-report hypertension

**Medication ever use (Cases/P-yrs)**	**Reference groups (Cases/P-yrs)**	**HR (95% CI)[1]**
ACEi (73/150,417)	Use of other anti-HT meds[2] (158/307,883)	0.96 (0.73-1.27)
	Non-use of any anti-HT meds (83/169,326)	0.89 (0.65-1.22)
β-blockers (50/113,231)	Use of other anti-HT meds[2] (181/345,070)	0.85 (0.62-1.16)
	Non-use of any anti-HT meds (83/169,326)	0.80 (0.57-1.14)
CCBs (98/154,604)	Use of other anti-HT meds[2] (133/303,696)	1.32 (1.01-1.71)
	Non-use of any anti-HT meds (83/169,326)	1.08 (0.81-1.44)
Diuretics (102/208,536)	Use of other anti-HT meds[2] (129/249,764)	0.90 (0.69-1.16)
	Non-use of any anti-HT meds (83/169,326)	0.86 (0.64-1.15)

[1] Competing-risk model adjusted for age at baseline, race/ethnicity (non-Hispanic White, Black, Hispanic, Asian or Pacific Islander, and American Indian/Alaska Native), BMI (<25, 25-<30, ≥30 kg/m^2), smoking status (never, former, current), alcohol consumption (< 3 drinks/dayand ≥ 3 drinks/day), and self-reported type 2 diabetes at baseline (yes or no).

[2] Use of other anti-HT medications (not including the one under study).

Supplementary Table S2. Use and duration of use of DHP- and non-DHP CCBs in association with risk of incident pancreatic cancer

Medication Use	Cases/P-yrs	HR (95% CI)[1]	HR (95% CI)[2]
Use of other anti-HT drugs[3]	193/411,065	1.00 (ref.)	1.00 (ref.)
Dihydropyridine CCBs ever use	78/131,592	1.22 (0.93-1.58)	1.19 (0.9-1.55)
Dihydropyridine CCBs use < 3 years	36/63,512	1.18 (0.82-1.68)	1.15 (0.81-1.65)
Dihydropyridine CCBs use ≥ 3 years	42/68,080	1.25 (0.89-1.74)	1.22 (0.87-1.70)
P trend		0.238	0.376
Use of other anti-HT drugs[3]	234/498,435	1.00 (ref.)	1.00 (ref.)
Non-dihydropyridine CCBs ever use	37/44,223	1.67 (1.18-2.36)	1.61 (1.14-2.28)
Non-dihydropyridine CCBs use <3	13/22,112	1.20 (0.69-2.10)	1.16 (0.66-2.03)
Non-dihydropyridine CCBs use ≥ 3	24/22,110	2.12 (1.39-3.22)	2.04 (1.34-3.11)
P trend		0.001	<0.001

Abbreviations: CCB=Calcium channel blocker; DHP= Dihydropyridine; P-yrs=Person-years; sRAGE=soluble receptor for advance glycation end products

[1] Adjusted for age at baseline, race/ethnicity (White (non-Hispanic), Black, Hispanic, Asian or Pacific Islander, and American Indian/Alaska Native), BMI (<25, 25-<30 and ≥30 kg/m^2), smoking status (never, former and current), alcohol consumption (< 3 drinks/day and ≥ 3 drinks/day), self-reported type 2 diabetes at baseline (yes or no), and self-reported hypertension at baseline (yes or no)

[2] Competing risks model with same covariates in (1).

[3] Use of other anti-HT drugs include all the rest anti-HT medications studied other than the one of interest

Supplementary Table S3. Baseline CCB use and risk of incident pancreatic cancer by potential effect modifiers in the WHI Study

Strata	Use status	Case/Person-years	HR (95% CI)[1]
BMI<25	Ever use of other anti-HT drugs[2]	25/83,745	1.00 (ref.)
	Ever CCB use	33/39,949	2.38 (1.42-4.03)
BMI 25-<30	Ever use of other anti-HT drugs[2]	49/113,587	1.00 (ref.)
	Ever CCB use	33/59,204	1.17 (0.76-1.83)
BMI≥30	Ever use of other anti-HT drugs[2]	66/133,233	1.00 (ref.)
	Ever CCB use	48/75,977	1.22 (0.84-1.78)
P-interaction		0.37	
WHR<0.8	Ever use of other anti-HT drugs[2]	47/134,784	1.00 (ref.)
	Ever CCB use	32/63,300	1.31 (0.84-2.10)
WHR≥0.8	Ever use of other anti-HT drugs[2]	93/195,817	1.00 (ref.)
	Ever CCB use	82/111,849	1.42 (1.06-1.92)
P-interaction		0.22	
No CVD	Ever use of other anti-HT drugs[2]	98/231,525	1.00 (ref.)
	Ever CCB use	62/106,015	1.33 (0.97-1.83)
CVD	Ever use of other anti-HT drugs[2]	34/79,141	1.00 (ref.)
	Ever CCB use	42/59,195	1.43 (0.91-2.28)
P-interaction		0.74	
No diabetes	Ever use of other anti-HT drugs[2]	124/22,516	1.00 (ref.)
	Ever CCB use	99/12,198	1.44 (1.10-1.89)
Diabetes	Ever use of other anti-HT drugs[2]	16/2,534	1.00 (ref.)
	Ever CCB use	15/1,902	1.26 (0.62-2.56)
P-interaction		0.72	

Abbreviations: anti-HT=anti-hypertensive; BMI=body mass index; CCB=calcium channel blocker; CI=confidence interval; CVD=cardiovascular disease; HR=hazard ratio; WHR=waist-to-hip ratio.

[1] Competing risk model adjusted for age at baseline, race/ethnicity (Non-Hispanic white, Black, Hispanic, Asian or Pacific Islander, and American Indian/Alaska Native), BMI (<25, 25-<30 and ≥30 kg/m^2, not adjusted when stratified by BMI), smoking status (never, former and current), alcohol consumption (< 3 drinks/dayand ≥ 3 drinks/day), self-reported type II diabetes at baseline (yes or no, not adjusted when stratified by diabetes status), and self-reported hypertension at baseline (yes or no).

[2] Ever use of anti-HT drugs other than the one of interest.

Supplementary Table S4. Duration of CCB use at baseline and risk of incident pancreatic cancer after excluding first 2 years of follow-up

Medication ever use (Cases/P-yrs)	Reference groups (Cases/P-yrs)	HR (95% CI)[1]
ACEi (61/143,372)	Use of other anti-HT meds[2] (178/398,445)	0.94 (0.70-1.27)
	Non-use of any anti-HT meds (526/1,453,512)	0.93 (0.68-1.28)
β-blockers (52/146,847)	Use of other anti-HT meds[2] (187/394,970)	0.76 (0.56-1.04)
	Non-use of any anti-HT meds (526/1,453,512)	0.80 (0.58-1.10)
CCBs (100/174,968)	Use of other anti-HT meds[2] (139/366,850)	1.44 (1.12-1.87)
	Non-use of any anti-HT meds (526/1,453,512)	1.24 (0.95-1.62)
Diuretics (106/248,018)	Use of other anti-HT meds[2] (133/293,799)	0.92 (0.71-1.19)
	Non-use of any anti-HT meds (526/1,453,512)	0.93 (0.72-1.20)

Abbreviations: anti-HT=anti-hypertensive; CCB=calcium channel blocker; CI=confidence interval; HR=hazard ratio.

[1] Competing risk model adjusted for age at baseline, race/ethnicity (Non-Hispanic white, Black, Hispanic, Asian or Pacific Islander, and American Indian/Alaska Native), BMI (<25, 25-<30 and ≥30 kg/m^2), smoking status (never, former and current), alcohol consumption (< 3 drinks/dayand ≥ 3 drinks/day), self-reported type II diabetes at baseline (yes or no), and self-reported hypertension at baseline (yes or no)

[2] Ever use of anti-HT drugs other than the one of interest

Supplementary Figure S1. Average serum sRAGE level by single anti-HT medication use among controls in the WHI Study (N =1,522)

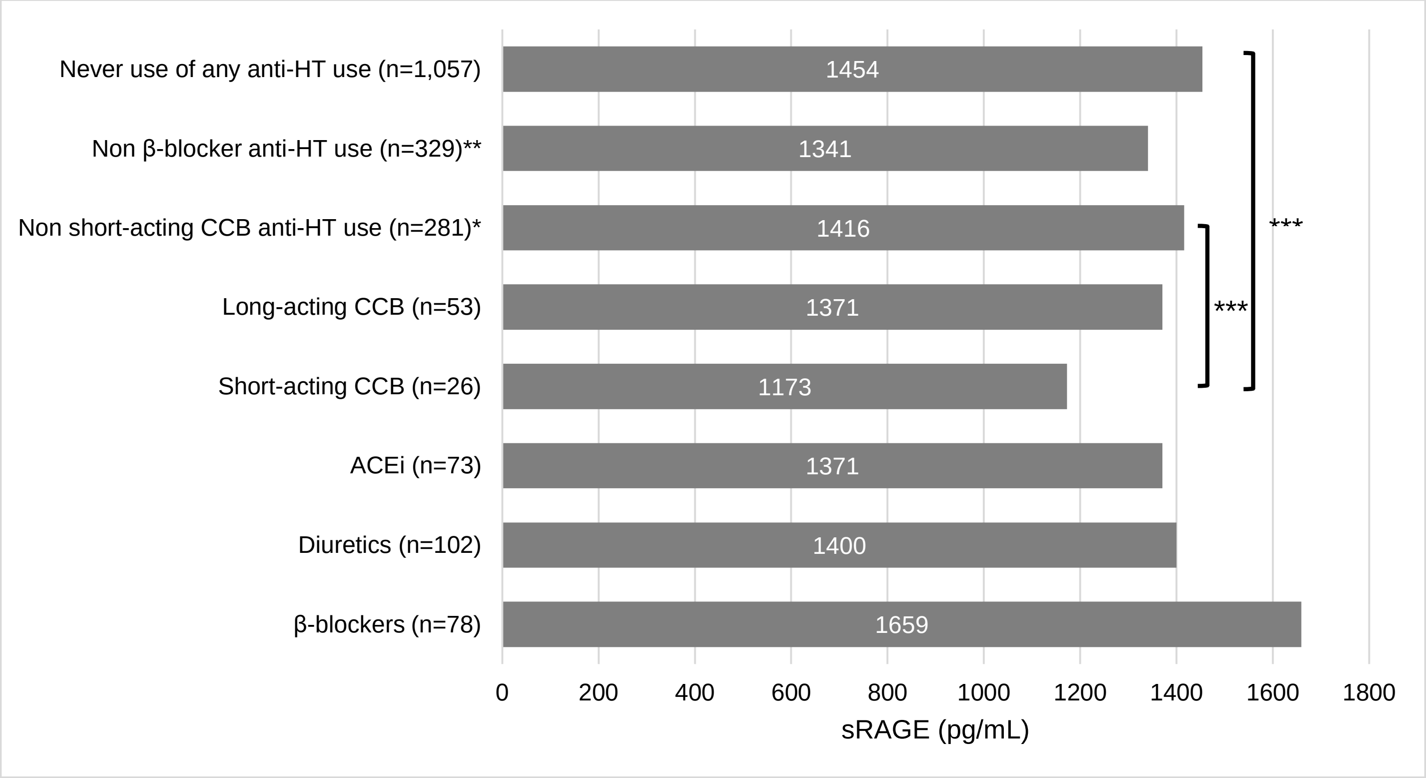

Abbreviations: ACEi=Angiotensin converting enzyme inhibitor; CCB=Calcium channel blocker; DHP=Dihydropyridine; NDHP=Non-dihydropyridine; sRAGE=soluble receptor for advanced glycation end products

* Including ever use of ACEi, diuretics, β-blockers, or DHP CCB

** Including ever use of ACEi, diuretics, β-blockers, or long-acting CCB

*** $P<0.05$, Sidak multiple comparison adjustment applied to compute *P*-value based on model adjusted for age at baseline, BMI (<25, 25-<30 and ≥30 kg/m^2), smoking status (never, former and current), alcohol consumption (< 3 drinks/day and ≥ 3 drinks/day), self-reported type 2 diabetes at baseline (yes or no) and self-reported hypertension at baseline (yes or no)
